# Classical Swine Fever in a Cuban Zone Intended for Eradication: Spatiotemporal Clustering and Risk Factors

**DOI:** 10.3389/fvets.2020.00038

**Published:** 2020-02-05

**Authors:** Osvaldo Fonseca-Rodríguez, Yosdany Centelles García, Pastor Alfonso Zamora, Edyniesky Ferrer-Miranda, Damarys de las Nieves Montano, Miriam Blanco, Yobani Gutiérrez, Paolo Calistri, Kleber Régis Santoro, María Irian Percedo

**Affiliations:** ^1^National Center for Animal and Plant Health (CENSA), OIE Collaborating Center for Reduction of the Risk of Disasters in Animal Health, Carretera de Jamaica y Autopista Nacional, San José de las Lajas, Cuba; ^2^Department of Epidemiology and Global Health, Umeå University, Umeå, Sweden; ^3^Federal Rural University of Pernambuco (UFRPE), Rua Dom Manuel de Medeiros, Recife, Brazil; ^4^Animal Health Division, Ministry of Agriculture, Pinar del Río, Cuba; ^5^Istituto Zooprofilattico Sperimentale dell'Abruzzo e del Molise “G. Caporale,” National Reference Centre for Epidemiology and Risk Analysis, Teramo, Italy

**Keywords:** classical swine fever, endemic, Cuba, outbreak, cluster, risk factors

## Abstract

Classical Swine Fever (CSF) is an endemic disease in Cuba, and an eradication strategy by zones is planned by the Official Veterinary Service. The aim of this study was to identify high-risk areas of CSF and the risk factors associated with the disease occurrence in the Pinar del Río province, one of the prioritized areas in the eradication strategy. The outbreak occurrence at district level was analyzed through a 7-year period (2009 to 2015). A high-risk cluster (RR = 5.13, 95% CI 3.49–7.56) was detected during the last 2 years of the study period in the eastern half of the province, with 38 out of 97 districts included. The rate of CSF-affected holdings had a significant increase during 2014–2015 and seems to have occurred mainly in the high-risk cluster area. Swine population density by district (heads/km^2^) and road length (km) by district were associated with the disease outbreak occurrence. These results provide new insights into the knowledge of the epidemiology of the disease in Cuban endemic conditions and can contribute to improving the control and the eradication strategy in this situation.

## Introduction

Classical swine fever (CSF) is a highly contagious viral disease caused by an RNA virus belonging to the genus *Pestivirus*, family *Flaviviridae* (1–3). The disease is endemic in several South and Central American countries, Cuba and the Hispaniola Island in the Caribbean region, Asia, and in some Eastern European countries (3–6).

CSF is a disease notifiable to the World Organization for Animal Health (OIE) and one of the most highly contagious animal diseases, with high morbidity and mortality in susceptible hosts, depending on the virus strain, immune status of the herd, and age of the pigs (7). Its outbreaks can cause enormous losses in naïve pig populations; it is considered a devastating disease for the pig industry throughout the world, concerning both economic and sanitary issues (8).

The CSF control strategy recommended in free regions^1^ like the European Union consists of culling all animals in infected herds, disinfection and movement restrictions of animals, materials, and people from premises within a given distance from the infected herd (10). In addition, the epidemiological forward and backward tracing of transmission contacts is required. These basic measures, however, might not always be sufficient to achieve a rapid disease eradication, and supplementary control measures, such as preemptive culling of animals near infected premises or emergency vaccination, may help to control the disease (11).

Taking into account the CSF endemism in Cuba, vaccination with a nationally produced live attenuated vaccine of the lapinized Chinese strain, quarantine of affected premises, and culling of the affected animals and their contacts, are among the most important actions taken to reduce the number of disease outbreaks (12). Small and medium-sized affected farms are always depopulated, while large ones are depopulated only when the outbreak seems out of control after having used the previously mentioned measures.

CSF has a recognized important economic impact (13) that in the case of developing countries, like Cuba, may also threaten food security. Cuban pork production is the main local source of meat and the most consumed source of animal origin protein with high traditional value. The complexity of CSF eradication and control can be tackled through strategies aiming at progressively eradicating the infection in various zones of the country.

In Cuba, the Veterinary Authority is implementing an eradication plan by zones and Pinar del Río province is one of the starting points. Pinar del Rio is a priority zone for CSF eradication due to both strategic and advantageous aspects. It is an important swine production area in the country, where also herds for swine genetic selection are located (14). Since it is the country's westernmost province, its border are mostly maritime and its land connections are limited to a few roads within a narrow border with only a province (Artemisa), where facilities for controlling animal movements are implemented.

The key points for the elimination of residual sources of infection in the province are the identification of possible geographical clusters of CSF and the evaluation of potential risk factors associated with the disease occurrence. The present paper describes the results of a study for the identification of spatiotemporal clusters and risk factors for CSF in Pinar del Rio province. This study aims at contributing to a better and deeper understanding of the epidemiology of CSF in this territory and leading to the formulation of better policies for its control via the implementation of more efficient control measures.

## Materials and Methods

### Study Area

Pinar del Río, the province selected for the present study, is the westernmost territory of Cuba with a total area of 8,850 km^2^. It is divided into 11 municipalities with 97 districts (or Popular Councils, the smallest territories, according to the Cuban political-administrative division). The mean area of the districts in Pinar del Río is 90.6 km^2^.

### Data and Data Source

The provincial department of the National Animal Health Division of the Ministry of Agriculture (DSA) provided data at district level on the swine population by productive sectors. The productive sectors considered were commercial farms and backyard (988 swine holdings in total). The available information about swine holdings is recorded in manuscript books (not digital). The data were compiled, organized, and carefully revised with an electronic spreadsheet (Microsoft Excel 2010). The reported information on 101 swine holdings affected by CSF (hereinafter “CSF outbreak”) in the province from 2009 to 2015 was also collected. Some potential explanatory variables were analyzed with the aim of identifying those factors associated with the disease occurrence at district level: (i) swine population (heads); (ii) swine population density (heads/km^2^); (iii) swine holdings density (holdings/km^2^); (iv) human population (inhabitants); (v) human population density (inhabitants/km^2^); (vi) distance (km) from the district centroid to the main road and access to the province; vii) road length (km)—number of km of roads in the district; (viii) road density (km/km^2^).

The swine population data is annually updated at municipality level by the DSA. However, for the study purpose, the swine population was computed at district level in 2012. Also, the only data about the human population at the same scale is from the 2012 national census report (15). Hence, we assumed that the animal and human populations remained stable over the study period.

The centroid of each district was calculated using Add Geometry Attributes tool (Centroid property); the distances from each district centroid to the main road (name of this road in Spanish: *Autopista Nacional*) were calculated using the Near tool and Tabulate intersection tool was used to calculate the length (km) of roads by district. These variables were calculated in ArcGIS 10.2.

### Statistical Analysis

#### Descriptive Statistics

Minimum, maximum, mean, and standard deviation were calculated to describe the explanatory variables by districts. The cumulative incidence of CSF outbreaks by year was also calculated.

#### Space-Time Analysis

A retrospective space-time scan statistics implemented in SaTScan™ 9.3 (16) was used to detect spatiotemporal clusters in the study area. The CSF affected holdings (outbreaks) by districts and year were considered cases and the non-affected holdings were defined as controls. Thus, the Bernoulli probability model and 9,999 replications were used to detect clusters with high rates of CSF outbreak occurrence, considering a maximum temporal windows size of 50% of the study period. The time aggregation by year was used.

The maximum radius of the circular scanning window is variable, so the analysis may be based on knowledge about the epidemiological characteristics of the disease (17). Also, the default option of including up to 50% of the population at risk could produce non-useful results from a practical point of view (18). Thus, the maximum radius of the spatial window in scan statistics was determined by Spatial autocorrelation (Global Moran's Index) using the Incremental Spatial Autocorrelation tool in ArcGIS 10.2. Moran's *I* allows to identify spatial clustering and distances at which spatial processes were most pronounced (19).

The Global Moran's *I* test identified the occurrence of a spatial cluster of CSF outbreaks. A peak (Z Score = 3.25, *p* < 0.001) around 37 km suggested that at this distance the most pronounced spatial clustering is produced. Thus, the radius of the circular scanning window without geographical overlap in SaTScan was established at 37 km.

#### Regression Analysis

The association between the explanatory variables and the outcome variable (number of CSF outbreaks) was assessed using a Generalized Linear Mixed Model (GLMM) with Poisson distribution. The analysis was carried out using the function glmmPQL (package MASS v.7.3-51.4) in R 3.5.0 (20).

The model used in our study was as follows:

$$\begin{array}{l} \text{Log}\left(\text{outcome}\right)=\text{intercept }+\text{ fixed effects}\\ \;\left(\text{independent variables}\right)\;+\text{ random effect}\\ \;\left(\text{Municipality}\right)\;+\text{ offset }\left(\text{log }\left(\text{Swine holdings}\right)\right) \end{array}$$

The model outcome is the rate of outbreaks by districts. The fixed effects were estimated by the inclusion of the previously mentioned explanatory variables in the model. Relative risk (RR) with 95% confidence intervals (95% CI) of fixed effects was estimated. To adjust by the number swine holdings per district this variable was included in the model as offset and the municipality was included as a random effect to control for the clustering effect.

In order to present the results on a more interpretable scale, the effect of some independent variables was rescaled. The variables “Distance from the district centroid to the main road in the province (km)” and “Road length (km)” were divided by 10; Swine population (heads), Human population (Inhabitants) and Human population density (inhabitants/km^2^) were divided by 1000; Road density (km/km^2^) was not modified, and the rest were divided by 100. Firstly, the association between the dependent and the independent variables was assessed through the univariate regression analysis. Variables with a *p-value* ≤ 0.2 were included in the multivariate model. The final model was adjusted manually by using the backward elimination procedure to remove the non-significant variables (*p* < 0.05).

## Results

**Figure 1:**
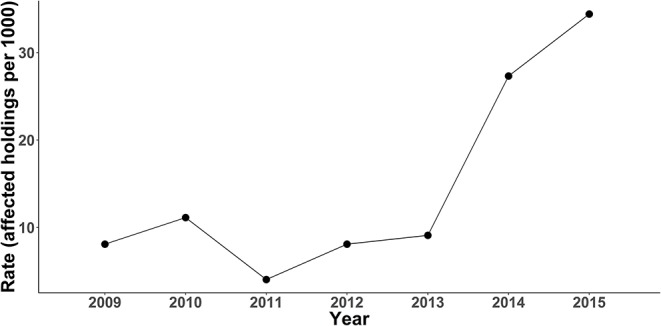
CSF outbreak rate by year in Pinar del Río province (2009–2015).

The swine population in Pinar del Río in 2012 was 226 444, 115 149 (50.86 %) in commercial herds and 111 295 (49.15 %) housed in backyard farms. Table 1 shows the descriptive statistics of the original (non-rescaled) explanatory variables, and in Figure 1 the annual rate of CSF outbreak per 1,000 swine holdings is shown. The annual rate was similar between 2009 to 2013; however, a drastic increment of cases occurred in the last 2 years of the study period.

**Table 1:** Descriptive result of explanatory variables at district level analyzed as potential risk factors for CSF outbreak occurrence in Pinar del Río province.

**Variables**	**Minimum**	**Maximum**	**Mean**	**Standard deviation**
Swine population (heads)	25	17328	2333.69	2152.1
Swine population density (heads/km^2^)	0.6	820.09	64.66	108.41
Swine holdings density (holdings/km^2^)	0	1.76	0.26	0.34
Human population (inhabitants)	1019	26912	5896.94	5029.48
Human population density (inhabitants/km^2^)	1.93	11500.85	524.6	1673.58
Distance from the district centroid to the main road (km)	0.09	88.66	26.6	21.33
Road length (km)	0	91.63	16.49	13.96
Road density (km/km^2^)	0	2.37	0.35	0.43

### Space-Time Cluster

**Figure 2:**
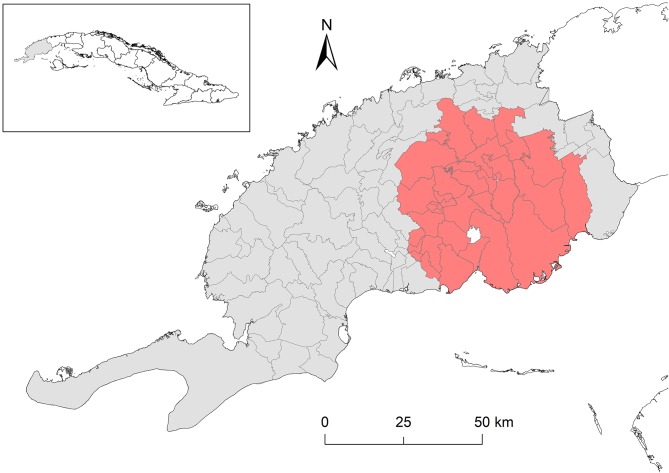
High-risk cluster of districts (highlighted) with CSF outbreak detected in Pinar del Río province (2009–2015). Complementary information is shown in Table 2.

A significant high-risk cluster (RR = 5.13, 95% CI 3.49–7.56) of CSF outbreaks at district level (Table 2 and Figure 2) was detected during the last 2 years (2014–2015) of the study period. The cluster included 38 districts of the eastern half of the province close to the neighboring province (Artemisa). The increasing number of outbreaks during these 2 years (Figure 1) occurred mainly in this area.

**Table 2:** Space-time cluster of CSF outbreaks in Pinar del Río province (2009–2015).

**Clusters**	**Start year**	**End year**	***p*-value**	**Number of districts**	**Observed CSF outbreak**	**Expected CSF outbreak**	**Relative risk (95% CI)**
1	2014	2015	0.001	38	44	13.20	5.13 (3.49–7.56)

### Risk Factors

Seven out of eight variables were associated with CSF occurrence in the univariate model according to the assumed cut-off (*p* ≤ 0.20) at this step. These variables were included in the multivariate model and two variables were retained. The swine population density was associated with the CSF outbreak. Thus, the RR of outbreak occurrence increases 1.27 (1.09–1.48) times for 100 swine/km^2^ in the districts. Also, the road length was associated with the disease occurrence, which increases 1.16 (1.04–1.29) times for 10 km of road. The outputs of the models are shown in Table 3.

**Table 3:** Univariate and multivariate mixed-effects model.

**Variables**	**Univariate models**			**Multivariate model**		
	***p*-value**	**RR**	**95%CI**	***p*-value**	**RR**	**95%CI**
Swine population (heads)	0.030	1.06	(1.01–1.11)			
Swine population density (heads/km^2^)	0.004	1.25	(1.08–1.45)	0.002	1.27	(1.09–1.48)
Swine holding density (holdings/km^2^)	0.412	0.74	(0.36–1.52)			
Human population (inhabitants)	0.007	1.06	(1.02–1.10)			
Human population density (inhabitants/km^2^)	0.106	1.11	(0.98–1.26)			
Distance from the district centroid to the main road (km)	0.022	0.84	(0.72–0.97)			
Road length (km)	0.029	1.14	(1.01–1.27)	0.010	1.16	(1.04–1.29)
Road density (km/km^2^)	0.121	1.52	(0.90–2.57)			

Risk factors identification for CSF outbreak occurrence in swine holdings in Pinar del Río province (2009–2015). Municipality − Random effect, Intra-class correlation (ICC) = 0.07.

*RR, Relative risk; CI, Confidence interval*.

## Discussion

The high-risk cluster observed in the eastern half of the province is an interesting result, because in this area there are important genetic and breeding pig farms. Breeding pig farms provide animals for fattening in other smaller farms by contract with the swine enterprise in the neighboring districts. Also, in genetic farms, pigs that are not selected as future parents for reproduction are also sent to other fattening farms. So, the pig movements in and out of the holdings in this area are more intensive than in other zones. Also, the animal density is higher in districts where intensive farms are settled due to the large number of housed animals.

The proximity of the high-risk cluster to the border with the Artemisa (Eastward) province could also be related to the transit of people and trade of live pigs and by-products from Pinar del Río to Artemisa and La Habana provinces, due to the lower prices in Pinar del Rio and the high pork demand of Cuban capital (21). The strict connections between pig breeders of Pinar del Río with those of Artemisa and Havana provinces may represent also an important risk factor for the introduction and dissemination of CSF in the study territory through fomites potentially contaminated or vehicles used for pig trade not adequately cleaned and disinfected. It is noteworthy that the Artemisa and Havana provinces have a significant worse epidemiological situation with regards to CSF than Pinar del Río. More outbreaks have been frequently reported in those neighboring provinces than in Pinar del Río; also, it must be considered that Pinar del Río is one of the provinces with the largest swine population in the country (approximately 14.36% of the national swine population). Thus, the large swine population and the relatively favorable epidemiological situation were taken into consideration to select the study area suitable as a territory to implement the CSF eradication campaign (21, 22).

The association between animal density and increased risk of transmission of infectious diseases has been already identified for CSF (23, 24) and for other diseases, such as African Swine Fever in Georgia (25), Foot and Mouth Disease in Tanzania (26) or Avian Influenza in Vietnam (27).

In addition, the presence of a greater road network can be associated with a higher risk of occurrence of CSF. Roads, railroads, and other transportation ways play a decisive role in infectious disease dissemination. The trade of live animals, by-products and transport of swine production items (feed, medicines, technical assistance, etc.) are based on vehicles, which can be an important mean of disease spreading (28, 29). Previous studies have considered roads as a risk factor for outbreak occurrence of infectious diseases such as African Swine Fever (30, 31) and Foot and Mouth Disease (32).

Another related aspect could be the use of undisinfected transport in the movement of the animals (33). Vehicles are an important component in the spreading of CSF, according to a study conducted by Martínez-López et al. (34) in Segovia, Spain, also in agreement with previous results carried out in the Netherlands (35, 36).

A significant number of swine in the study territory (~50%) and in Cuba (>50%) are housed in backyard holdings that do not follow the appropriated biosecurity rules such as sanitary barriers, cleaning, disinfection, vaccination, and an appropriated food storage, and sometimes the pigs are fed on swill containing pig by-products (6). Pigs raised under those conditions could contribute to the maintenance of the circulating viruses in the swine population. Also, despite unvaccinated animal movement being forbidden, the illegal movement of unvaccinated backyard animals persists, because it is very hard to control. Uncontrolled pig movements of small backyard producers could play an important role in diseases spreading (37) and could be a very important factor in the province.

This constant flow could raise the risk of CSF occurrence in Pinar del Río because its sanitary situation was historically favorable. The positive evolution of the CSF until 2002 was key to choose Pinar del Río to start the CSF eradication strategy by zones (14), in contrast with the bordering province of Artemisa (22). The spread of diseases by livestock movement along the borderlines of geographically contiguous regions with different epidemiological statuses is an essential factor that can affect the disease control, eradication, or disease-free status (38, 39).

This study focuses mainly on the detection of spatiotemporal clusters and the analysis of potential risk factors for CSF outbreaks occurrence considering its spatial distribution and explanatory variables. However, the non-availability of data about other variables made it impossible to include in the analysis many other potentially important factors, such as the number of visits to the farms, variations in total swine population by year and by different animal categories, vaccination coverage at district level, and animal movement, among others, which is an important limitation of this study. Future investigations could consider those factors to provide a more comprehensive assessment of the different factors that may influence both the occurrence and persistence of CSF at sub-district or farm level.

The identification of risk factors and the detection of areas where affected holdings are spatially more concentrated can highlight the territories where the sanitary measures must be reinforced to improve the disease control. It will be important to increase the awareness and risk perceptions of pig producers in all sectors using educational activities focused on biosecurity, pig movement control, vehicle disinfection, and vaccination, among others.

The methods used allowed to obtain interesting and useful results, which contributed to a better understanding of the CSF distribution in our scenario. These tools have shown the spatiotemporal distribution and risk factors of the disease and can be applied in the epidemiological studies in other provinces of the country to improve the control and eradication strategies at national level. However, this depends on data availability, which is an important limiting factor for this type of study.

In conclusion, the disease has space-time clustering and one high-risk area was identified; furthermore, two risk factors of outbreak occurrence were detected. The epidemiological situation showed a worsening over time with the increasing number of CSF outbreaks in the last two years of the study period, being significantly higher in the eastern part of the province. The policymakers should consider these findings in other to improve the CSF control program in Pinar del Río and to reinforce the sanitary measures in this territory. Moreover, these results can contribute to support the strategies in the CSF eradication campaign in Cuba, with stricter regulations of animal movement in districts at high risk, increasing the epidemiological risk-based surveillance and the vaccination prioritization in the high-risk areas.

## Data Availability Statement

The datasets generated for this study will not be made publicly available. The datasets used in this study belong to the National Animal Health Division of the Ministry of Agriculture (DSA), Cuba. The authors do not have authorization to publish the datasets.

## Ethics Statement

The research was performed with secondary and aggregated data. No study (experiment) with human or animal subjects was performed by any of the authors. Also, no private information from owners or farms was used or revealed.

## Author Contributions

OF-R performed data analysis and manuscript drafting. YC, PA, EF-M, DM, and MB critically read the manuscript, suggested corrections, and provided intellectual inputs. MB, YG, EF-M, and OF-R performed data collection. PC, KS, and MP provided supervision and contributed to the epidemiological studies. All authors reviewed and approved the final manuscript.

### Conflict of Interest

The authors declare that the research was conducted in the absence of any commercial or financial relationships that could be construed as a potential conflict of interest.

## Footnotes

^1^Region where the swine population is free of the infection and no vaccination against the disease is applied in domestic and captive wild pigs during the past 12 months (9).
